# A Novel Gaze Tracking Method Based on the Generation of Virtual Calibration Points

**DOI:** 10.3390/s130810802

**Published:** 2013-08-16

**Authors:** Ji Woo Lee, Hwan Heo, Kang Ryoung Park

**Affiliations:** Division of Electronics and Electrical Engineering, Dongguk University, 26 Pil-dong 3-ga, Jung-gu, Seoul 100-715, Korea; E-Mails: ljwgs@dongguk.edu (J.W.L.); gjghks@dgu.edu (H.H.)

**Keywords:** gaze tracking, multi-geometric transforms, multilayer perceptron, virtual calibration points

## Abstract

Most conventional gaze-tracking systems require that users look at many points during the initial calibration stage, which is inconvenient for them. To avoid this requirement, we propose a new gaze-tracking method with four important characteristics. First, our gaze-tracking system uses a large screen located at a distance from the user, who wears a lightweight device. Second, our system requires that users look at only four calibration points during the initial calibration stage, during which four pupil centers are noted. Third, five additional points (virtual pupil centers) are generated with a multilayer perceptron using the four actual points (detected pupil centers) as inputs. Fourth, when a user gazes at a large screen, the shape defined by the positions of the four pupil centers is a distorted quadrangle because of the nonlinear movement of the human eyeball. The gaze-detection accuracy is reduced if we map the pupil movement area onto the screen area using a single transform function. We overcame this problem by calculating the gaze position based on multi-geometric transforms using the five virtual points and the four actual points. Experiment results show that the accuracy of the proposed method is better than that of other methods.

## 1. Introduction

Gaze-tracking technology is used to detect a user's gaze position in many applications, such as computer interfaces for the disabled, medical care, rehabilitation, and virtual reality [1–3]. Two approaches for gaze tracking exist: the wearable type and the remote type. The wearable type requires a user to wear a device that includes a camera and a near-infrared (NIR) light illuminator. Various types of devices can be used, such as a helmet or a pair of glasses [4–6], which do not require adjustments for head movements, because the device follows the user's head movements. However, when calculating the gaze position on a screen, tracking the head movements requires additional NIR illuminators in the four corners of the screen or an additional camera [4–6]. With the remote-type method, the user does not need to wear a device, because a remote camera captures an image of the user's eye, which is more convenient for the user [7,8]. However, additional cameras or expensive pan-tilt devices are required to capture eye images when users move their head.

Previous studies of gaze tracking can be classified into 2D- or 3D-based gaze-tracking methods. The 2D-based gaze-tracking methods use a simple mapping function between the pupil's position and the gaze position on the screen [4–6,9–11]. In contrast, the 3D-based gaze-tracking methods calculate the gaze position based on a 3D eyeball model [12,13]. In general, the 3D-based method is more accurate than the 2D-based method, but it requires the complex calibration of stereo cameras or multiple light sources.

In all previous studies on gaze tracking, an initial user calibration stage was required for more accurate gaze estimation. During the user calibration, the user needs to gaze at reference positions on a screen. In general, the accuracy of a gaze-tracking system tends to increase with the number of reference points, but this can be highly inconvenient for the user. To minimize the user inconvenience, NIR illuminators are attached to the four corners of the monitor and the system requires that users view only one position during Kappa calibration [5,6,13]. Table 1 provides a summary of the number of calibration points required by previous gaze-tracking methods and our proposed method.

To minimize the number of calibration points while maintaining the accuracy of gaze tracking, we propose a new gaze-tracking method based on the generation of virtual calibration points. In this study, we adopted a wearable gaze-tracking method to avoid the use of bulky panning and tilting devices while allowing natural head movements with a large display. We also used a 2D-based method in this study to reduce the complex calibrations of stereo cameras or multiple light sources that are required by 3D-based gaze-tracking methods.

In a previous study, Memmert used an eye-tracking system with a large screen (3.2 &times 2.4 m) [26]. Agustin *et al.* also proposed a gaze-tracking system that used a large screen [22]. With a large screen, the shape defined by the four pupil center positions (the top-left, top-right, bottom-left, and bottom-right corners of the screen) is a distorted quadrangle rather than a rectangle because of the nonlinear movement of the 3D eyeball. Thus, the gaze-detection accuracy is reduced if we map the pupil movement area onto the screen area using a single transform function. We overcame this problem by calculating the final gaze position based on multi-geometric transforms. The remainder of this paper is organized as follows: in Section 2, we explain the proposed method. The experiment results and conclusions are presented in Sections 3 and 4, respectively.

## 2. Proposed Gaze-Tracking Method

### 2.1. Overview of the Proposed Method

**Figure 1.**
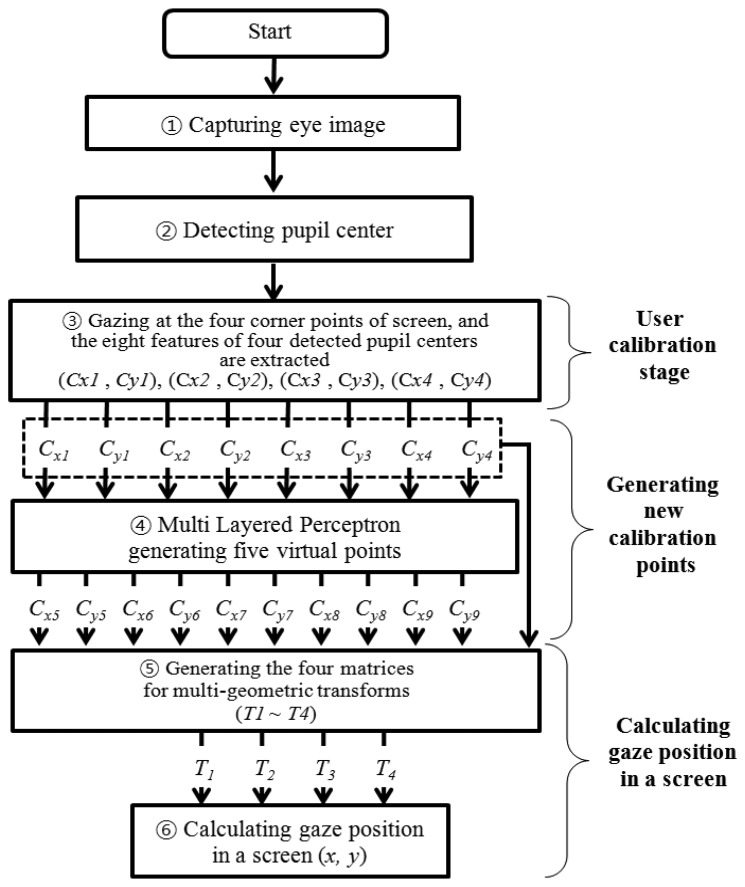
Flowchart of the proposed method.

**Figure 2.**
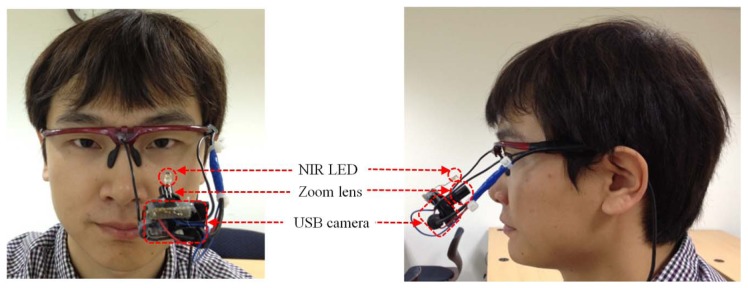
Proposed gaze-tracking device.

Figure 1 provides an overview of the proposed gaze-tracking method. First, a user's eye image is captured with a camera using the NIR illuminator in the device, as shown in Figure 2. The image is not affected by external visible light, because an IR-passing filter attached to the camera rejects visible light [10,11]. Second, the captured NIR eye image is processed, and the pupil center is detected (Section 2.3). Third, the user is required to gaze at points near the four corners of the screen during the user-calibration stage. The eight feature values of the four detected pupil centers are then extracted. (Section 2.4). Fourth, the eight extracted values are used as the inputs for training the multilayer perceptron (MLP). The MLP has a linear kernel, and it generates five additional points or virtual pupil centers as outputs (Section 2.5). Finally, the five generated points (virtual pupil centers) and the four actual points (detected pupil centers) are used to calculate the final gaze position (*x*, *y*) on a screen, based on multi-geometric transforms (Section 2.6).

### 2.2. Proposed Gaze-Tracking Device

In this study, we developed a gaze-tracking method that uses a wearable-type device. As shown in Figure 2, the device is comprised of a small universal serial bus (USB) camera and an NIR LED (light emitting diode) [10,11]. The USB camera is a Logitech WebCam C600 [27]. The NIR-passing (visible light rejection) filter is included in the camera, and an additional zoom lens is attached to the camera's built-in lens [10,11]. Thus, the camera can capture the magnified NIR eye image unaffected by external visible light. The Z distance between the camera lens and the eye is about 8 cm. The detailed specifications are as follows:

- NIR LED
  Wavelength: 850 nm
- Zoom lens
  Magnification ratio: &times 2.34
- USB camera
  Product Name: Logitech WebCam C600 [27]
  Spatial Resolution: 640 × 480 pixels (CMOS sensor)
  Frame rate: 30 fps

### 2.3. Detecting the Pupil Center

**Figure 3.**
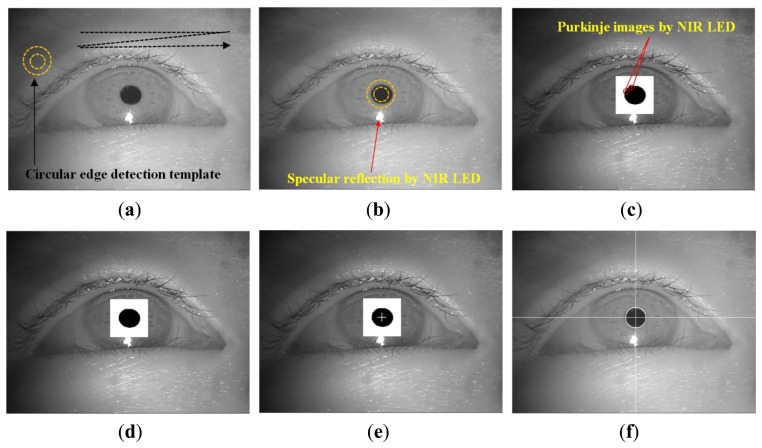
Procedure for detecting the pupil center: (**a**) detection of a circular edge in an eye image, (**b**) result of pupil detection based on circular edge detection, (**c**) local binarization and generation of Purkinje images in the pupil area, (**d**) removal of the Purkinje images by morphological closing, (**e**) detection of the pupil region center, and (**f**) resultant image of the pupil center detection.

In Step 2 of Figure 1, The circular edge detection (CED), local binarization, morphological closing, and geometric center calculation are performed sequentially to detect the pupil region in the NIR eye image, as shown in Figure 3 [6,10,11,28]. First, two scalable concentric circles (external and internal circles) are moved together within the whole image, as shown Figure 3a. The pupil center is determined to be the position where the average of sum of pixel difference between the outer circle and the inner circle is maximized, as shown in Figure 3b. The detected pupil region is used to define a rectangular region on which local binarization is performed, as shown in Figure 3c. The threshold value for the local binarization is determined using the p-tile method [29]. The NIR LED used in our device (Figure 2) generates two types of reflections: specular reflections and Purkinje images. As shown in Figure 3b, specular reflections are produced on the corneal surface and are referred to as the first Purkinje image [11]. The specular reflections are not included in the pupil area because of the relative positions of the NIR LED and the user's eye, as shown in Figures 3b and c; hence, they are not used to detect the pupil center in our study.

Additional reflections are produced by the NIR LED, as shown in Figure 3c. These reflections occur on the posterior surface of the cornea and the anterior and posterior surfaces of the lens. These are referred to as the second, third, and fourth Purkinje images, respectively [11]. In our eye images, two Purkinje images were found in the pupil area and one in the iris area, as shown in Figure 3c. To determine the accurate pupil center, the Purkinje images in the pupil area were filled in using morphological closing, as shown in Figure 3d [30]. Finally, the geometric center position of the black pixels in the pupil region is calculated as the pupil center, as shown in Figure 3e, and the final detection result is shown in Figure 3f.

### 2.4. User Calibration by Gazing at the Four Corners of a Screen

**Figure 4.**
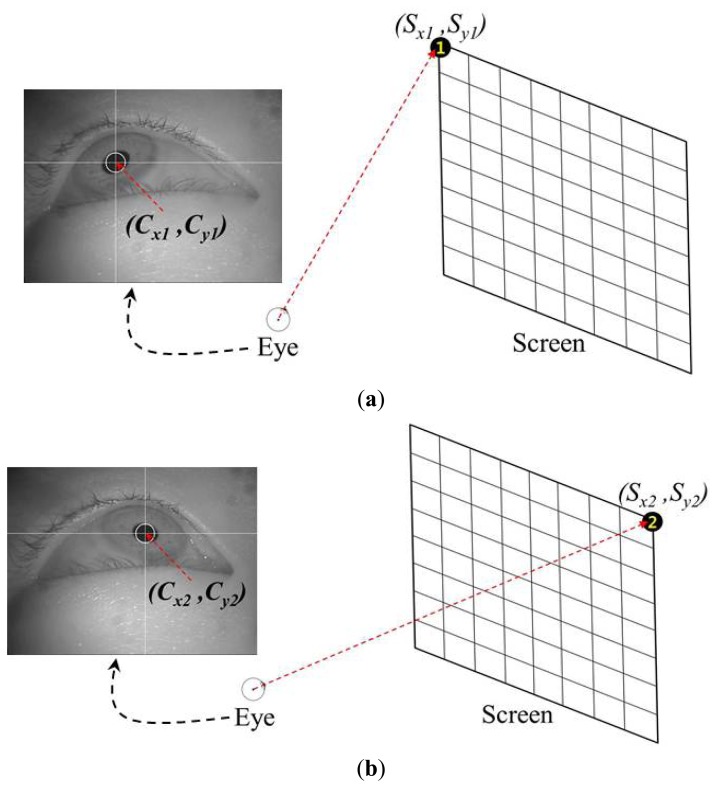

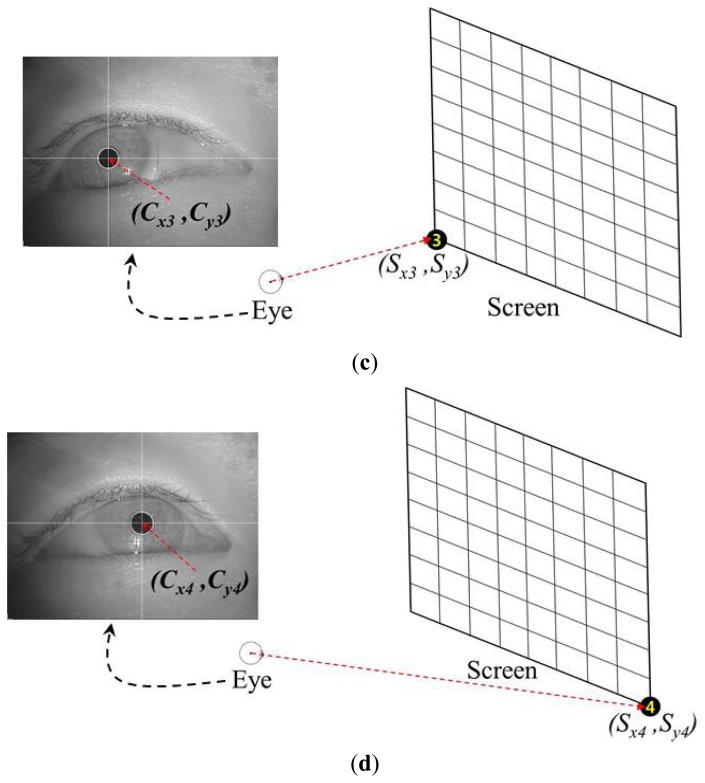
User-dependent calibration stage where a user gazes at four corners of a screen: (**a**) gazing at the top-left corner, (**b**) gazing at the top-right corner, (**c**) gazing at the bottom-left corner, and (**d**) gazing at the bottom-right corner.

Most conventional gaze-tracking systems require an initial user-dependent calibration procedure. In the present study, a user is requested to gaze at the four corners of a screen, as shown in Figure 4. When the user gazes at the four reference points, the four center positions of the user's pupil [(*C_x1_*, *C_y1_*), (*C_x2_*, *C_y2_*), (*C_x3_*, *C_y3_*), and (*C_x4_*, *C_y4_*)] are obtained. Figure 4a–d show the four eye images and the pupil center positions when a user gazes at the top-left, top-right, bottom-left, and bottom-right reference points, respectively. These eight values [(*C_x1_*, *C_y1_*), (*C_x2_*, *C_y2_*), (*C_x3_*, *C_y3_*), and (*C_x4_*, *C_y4_*)] are used in the next step to estimate the five virtual points using the MLP algorithm.

### 2.5. Generating Five Virtual Points Using the MLP Algorithm

**Figure 5.**
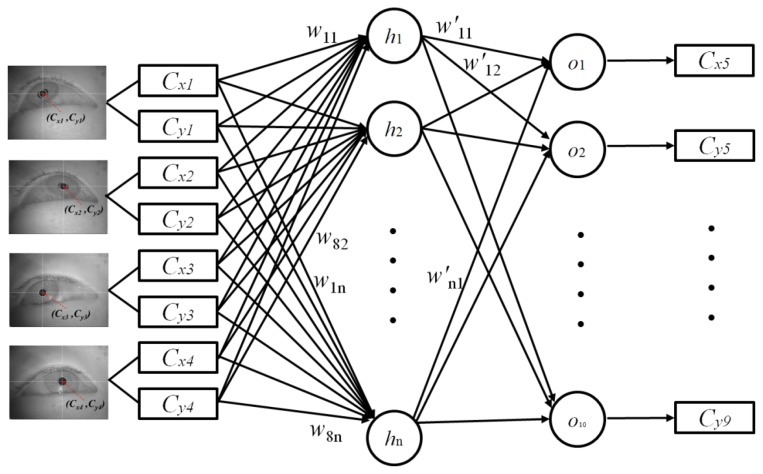
MLP to estimate the five virtual points in the pupil center.

As explained in Section 2.4, the four center positions of the user's pupil [(*C_x1_*, *C_y1_*), (*C_x2_*, *C_y2_*), (*C_x3_*, *C_y3_*), and (*C_x4_*, *C_y4_*)] are obtained during the user-calibration stage. The eight values [(*C_x1_*, *C_y1_*), (*C_x2_*, *C_y2_*), (*C_x3_*, *C_y3_*), and (*C_x4_*, *C_y4_*)] are used as the inputs for the MLP to estimate the five virtual points of the pupil centers [(*C_x5_*, *C_y5_*), (*C_x6_*, *C_y6_*), (*C_x7_*, *C_y7_*), (*C_x8_*, *C_y8_*), and (*C_x9_*, *C_y9_*)], as shown in Figure 5. A back-propagation algorithm is used to train the MLP [31], which has eight input and ten output nodes.

Additional user calibration points, *i.e.*, (*C_x5_*, *C_y5_*), (*C_x6_*, *C_y6_*), (*C_x7_*, *C_y7_*), (*C_x8_*, *C_y8_*), and (*C_x9_*, *C_y9_*), are obtained, and one of the points (*C_x5_*) can be represented using Equations (1–4) [11]:

(1) $$C_{x5}=\text{func}2({w^{\prime }}_{11}\cdot O\_h_{1}+{w^{\prime }}_{21}\cdot O\_h_{2}+{w^{\prime }}_{31}\cdot O\_h_{3}+\cdots +{w^{\prime }}_{n1}\cdot O\_h_{n})$$

where *O*_*h*_i_ is the output value of the hidden node (*h*_i_) and *w*'_i1_ is the weight between the hidden node (*h*_i_) and the output node (*o*_1_). Various kernel functions can be used for the hidden and output nodes, such as linear or sigmoid functions. For a linear function, Equation (1) can be represented as follows:

(2) $$C_{x5}={w^{\prime }}_{11}\cdot O\_h_{1}+{w^{\prime }}_{21}\cdot O\_h_{2}+{w^{\prime }}_{31}\cdot O\_h_{3}+\cdots +{w^{\prime }}_{n1}\cdot O\_h_{n}$$

*O*_*h_1_*, *O*_*h_2_*, *O*_*h_3_*, …, *O*_*h_n_* can also be represented as follows:

(3) $$\begin{array}{c} O\_h_{1}=\text{func}1(C_{x1}\cdot w_{11}+C_{y1}\cdot w_{21}+C_{x2}\cdot w_{31}+\cdots +C_{y4}\cdot w_{81})\\ O\_h_{2}=\text{func}1(C_{x1}\cdot w_{12}+C_{y1}\cdot w_{22}+C_{x2}\cdot w_{32}+\cdots +C_{y4}\cdot w_{82})\\ O\_h_{3}=\text{func}1(C_{x1}\cdot w_{13}+C_{y1}\cdot w_{23}+C_{x2}\cdot w_{33}+\cdots +C_{y4}\cdot w_{83})\\ \vdots \\ O\_h_{n}=\text{func}1(C_{x1}\cdot w_{1n}+C_{y1}\cdot w_{2n}+C_{x2}\cdot w_{3n}+\cdots +C_{y4}\cdot w_{8n}) \end{array}$$

where *func*1(·) is the kernel function of the hidden node (*h_i_*). After replacing *O_h*_1_, *O_h*_2_, *O_h*_3_, …, *O_h*_n_ in Equation (1) using Equation (3)*C_x5_* can be represented as follows:

(4) $$\begin{array}{c} C_{x5}=\text{func}2(w\prime_{11}\cdot \text{func}1(C_{x1}\cdot w_{11}+C_{y1}\cdot w_{21}+C_{x2}\cdot w_{31}+\cdots +C_{y4}\cdot w_{81})\\ +w\prime_{21}\cdot \text{func}1(C_{x1}\cdot w_{12}+C_{y1}\cdot w_{22}+C_{x2}\cdot w_{32}+\cdots +C_{y4}\cdot w_{82})\\ +w\prime_{31}\cdot \text{func}1(C_{x1}\cdot w_{13}+C_{y1}\cdot w_{23}+C_{x2}\cdot w_{33}+\cdots +C_{y4}\cdot w_{83})\\ \vdots \\ +w\prime_{n1}\cdot \text{func}1(C_{x1}\cdot w_{1n}+C_{y1}\cdot w_{2n}+C_{x2}\cdot w_{3n}+\cdots +C_{y4}\cdot w_{8n})) \end{array}$$

**Figure 6.**
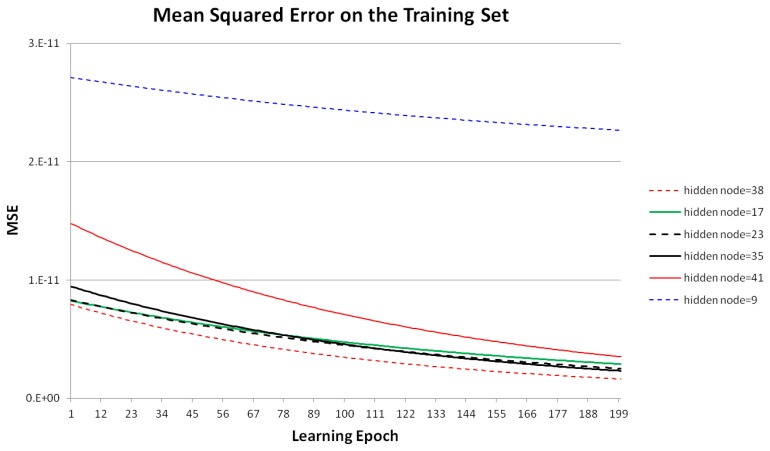
Mean squared errors (MSE) of MLP training using different numbers of hidden nodes.

Figure 6 shows the MSE using different numbers of hidden nodes with the training data, given eight input and ten output nodes in the MLP, as shown in Figure 5. The MSE decreased as the learning epoch increased during MLP training. In this experiment, we compared the MSE on the training set according to the numbers of hidden nodes from 1–50. Based on the minimum MSE in the experiment results, we selected 38 as the optimal number of hidden nodes. To simplify the graph, Figure 6 shows only the cases where the numbers of hidden nodes are 9, 17, 23, 35, 38 (optimal), and 41.

**Figure 7.**
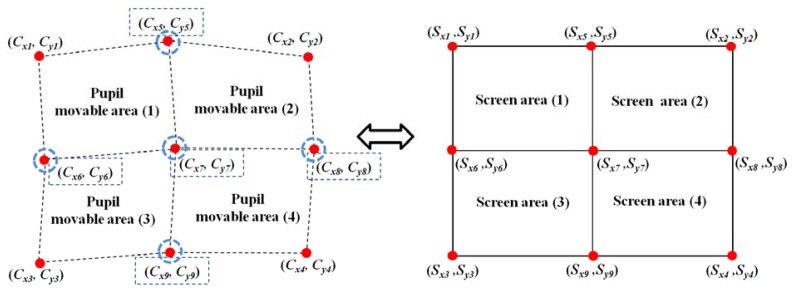
Four pupil movable areas (defined by the four actual pupil centers and five virtual (generated) pupil centers) and the corresponding screen areas.

The left part of Figure 7 shows examples of the four pupil movable areas defined using the four actual pupil centers [(*C_x1_*, *C_y1_*), (*C_x2_*, *C_y2_*), (*C_x3_*, *C_y3_*), and (*C_x4_*, *C_y4_*)] and the five virtual (generated) pupil centers [(*C_x5_*, *C_y5_*), (*C_x6_*, *C_y6_*), (*C_x7_*, *C_y7_*), (*C_x8_*, *C_y8_*), and (*C_x9_*, *C_y9_*)].

For example, Pupil Movable Area 1 is defined by (*C_x1_*, *C_y1_*), (*C_x5_*, *C_y5_*), (*C_x6_*, *C_y6_*), and (*C_x7_*, *C_y7_*). The right part of Figure 7 shows the four screen areas corresponding to each pupil movable area. For example, Pupil Movable Area 1 corresponds to Screen Area 1. Based on these relationships between the pupil movable areas and the screen areas, multi-geometric transforms are obtained, and the final gaze position is calculated. Detailed explanations are provided in the following Section.

### 2.6. Calculating Final Gaze Position using Multi-geometric Transforms

As shown in Figure 7, four relationships are defined between the four pupil movable areas and the four screen areas after the user-dependent calibration stage is completed; for example, the relationship between Pupil Movable Area 1 and Screen Area 1. The pupil movable area and the screen area are a distorted quadrangle and a rectangle, respectively, as shown in Figure 7; hence, each relationship can be determined as a mapping function. In general, 1st-order or 2nd-order polynomials are used as the mapping function, as shown in Equations (5) and (6).

With the 1st-order polynomial function, the relationship between the coordinates of the pupil center (*C_x_*, *C_y_*) and the calculated position on the screen (*S_x_*, *S_y_*) is as follows:

(5) $$\begin{array}{c} S_{x}=a\cdot C_{x}+b\cdot C_{y}+c\cdot C_{x}\cdot C_{y}+d\\ S_{y}=e\cdot C_{x}+f\cdot C_{y}+g\cdot C_{x}\cdot C_{y}+h \end{array}$$

As shown in Equation (5), the 1st-order polynomial function includes eight parameters, which consider the 2D factors of rotation, translation, scaling, parallel inclining, and distortion between (*C_x_*, *C_y_*) and (*S_x_*, *S_y_*) [32]. This is referred to as a geometric transform mapping function [10,11].

As shown in Equation (6), the 2nd-order polynomial function includes the 2nd-order parameters, in addition to the parameters of the 1st-order polynomial function [20,21]:

(6) $$\begin{array}{c} S_{x}=a\cdot {C_{x}}^{2}+b\cdot {C_{y}}^{2}+c\cdot C_{x}+d\cdot C_{y}+e\cdot C_{x}\cdot C_{y}+f\\ S_{y}=g\cdot {C_{x}}^{2}+h\cdot {C_{y}}^{2}+i\cdot C_{x}+j\cdot C_{y}+k\cdot C_{x}\cdot C_{y}+l \end{array}$$

Equations (5) and (6) can be represented using a transform matrix, as shown in Equations (7) and (8):

(7) $$\left[\begin{array}{c} S_{x}\\ \begin{array}{c} S_{y}\\ \begin{array}{c} 0\\ 0 \end{array} \end{array} \end{array}\right]=\left[\begin{array}{cccc} a & b & c & d\\ e & f & g & h\\ 0 & 0 & 0 & 0\\ 0 & 0 & 0 & 0 \end{array}\right]\;\left[\begin{array}{c} C_{x}\\ \begin{array}{c} C_{y}\\ \begin{array}{c} C_{x}\cdot C_{y}\\ 1 \end{array} \end{array} \end{array}\right]$$

(8) $$\left[\begin{array}{c} S_{x}\\ \begin{array}{c} S_{y}\\ \begin{array}{c} 0\\ \begin{array}{c} 0\\ 0\\ 0 \end{array} \end{array} \end{array} \end{array}\right]=\left[\begin{array}{cccccc} a & b & c & d & e & f\\ g & h & i & j & k & l\\ 0 & 0 & 0 & 0 & 0 & 0\\ 0 & 0 & 0 & 0 & 0 & 0\\ 0 & 0 & 0 & 0 & 0 & 0\\ 0 & 0 & 0 & 0 & 0 & 0 \end{array}\right]\;\left[\begin{array}{c} \begin{array}{c} {C_{x}}^{2}\\ {C_{y}}^{2}\\ C_{x} \end{array}\\ \begin{array}{c} C_{y}\\ \begin{array}{c} C_{x}\cdot C_{y}\\ 1 \end{array} \end{array} \end{array}\right]$$

**Figure 8.**
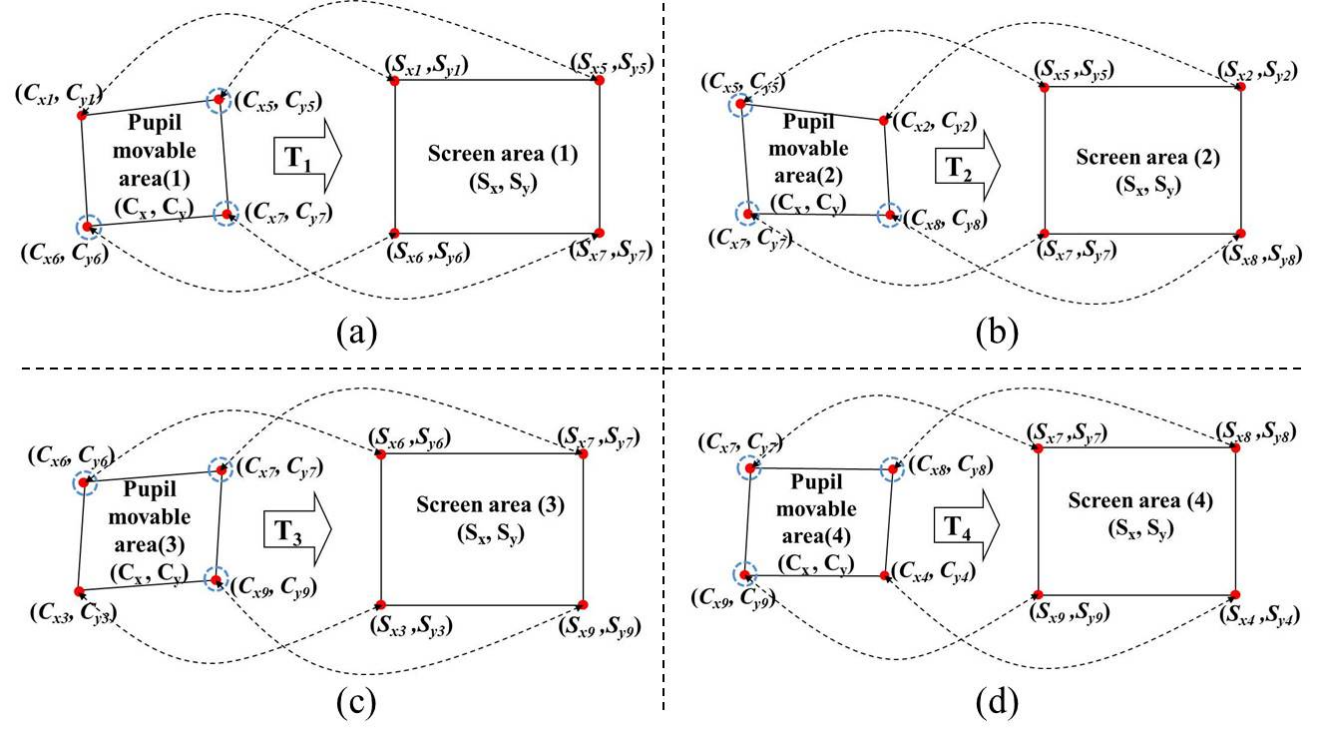
Four mapping transforms between the four pupil movable areas and four screens: (**a**) between Pupil Movable Area 1 and Screen Area 1; (**b**) between Pupil Movable Area 2 and Screen Area 2; (**c**) between Pupil Movable Area 3 and Screen Area 3; and (**d**) between Pupil Movable Area 4 and Screen Area 4.

In this study, we use multi-geometric transformations (multiple 1st-order polynomial functions) with the nine calibration points (the four actual pupil centers, *i.e.*, (*C_x1_*, *C_y1_*), (*C_x2_*, *C_y2_*), (*C_x3_*, *C_y3_*), and (*C_x4_*, *C_y4_*), and the five virtual (generated) pupil centers, *i.e.*, (*C_x5_*, *C_y5_*), (*C_x6_*, *C_y6_*), (*C_x7_*, *C_y7_*), (*C_x8_*, *C_y8_*), and (*C_x9_*, *C_y9_*), shown in Figure 7. Four mapping transforms (**T_1_**, **T_2_**, **T_3_**, and **T_4_**) are defined between the four pupil movable areas and four screen areas, as shown in Figure 8.

As shown in Figure 8a, **T_1_** is the mapping transform matrix between Pupil Movable Area 1 and Screen Area 1. Using the training data, **T_1_** can be obtained in advance by multiplying **S_1_**′ and the inverse matrix of **C_1_**′ in Equation (9) [10,11].

(9) $$\begin{array}{ccc} {\text{S}}_{1}\prime & {\text{T}}_{1} & {\text{C}}_{1}\prime \\ \left[\begin{array}{c} S_{x1}\\ \begin{array}{c} S_{y1}\\ \begin{array}{c} 0\\ 0 \end{array} \end{array} \end{array}\begin{array}{c} S_{x5}\\ \begin{array}{c} S_{y5}\\ \begin{array}{c} 0\\ 0 \end{array} \end{array} \end{array}\begin{array}{c} S_{x6}\\ \begin{array}{c} S_{y6}\\ \begin{array}{c} 0\\ 0 \end{array} \end{array} \end{array}\begin{array}{c} S_{x7}\\ \begin{array}{c} S_{y7}\\ \begin{array}{c} 0\\ 0 \end{array} \end{array} \end{array}\right]= & \left[\begin{array}{ccc} a_{1} & b_{1} & \begin{array}{cc} c_{1} & d_{1} \end{array}\\ e_{1} & f_{1} & \begin{array}{cc} g_{1} & h_{1} \end{array}\\ \begin{array}{c} 0\\ 0 \end{array} & \begin{array}{c} 0\\ 0 \end{array} & \begin{array}{c} \begin{array}{cc} 0 & 0 \end{array}\\ \begin{array}{cc} 0 & 0 \end{array} \end{array} \end{array}\right] & \left[\begin{array}{c} C_{x1}\\ \begin{array}{c} C_{y1}\\ \begin{array}{c} C_{x1}\cdot C_{y1}\\ 1 \end{array} \end{array} \end{array}\begin{array}{c} C_{x5}\\ \begin{array}{c} C_{y5}\\ \begin{array}{c} C_{x5}\cdot C_{y5}\\ 1 \end{array} \end{array} \end{array}\begin{array}{c} C_{x6}\\ \begin{array}{c} C_{y6}\\ \begin{array}{c} C_{x6}\cdot C_{y6}\\ 1 \end{array} \end{array} \end{array}\begin{array}{c} C_{x7}\\ \begin{array}{c} C_{y7}\\ \begin{array}{c} C_{x7}\cdot C_{y7}\\ 1 \end{array} \end{array} \end{array}\right] \end{array}$$

During the testing stage, if the position vector of the detected pupil center belongs to the quadrangle of Pupil Movable Area 1, the **T_1_** matrix in Equation (9) is selected and the gaze position vector on the screen is calculated by multiplying **T_1_** and the position vector of the detected pupil center [10,11]. By the same method, **T_2_**, **T_3_**, and **T_4_** of Figure 8b, c, and d are obtained, and the gaze position vector on the screen is also calculated.

Previous studies [10,11] also used the 1st-order polynomial function (geometric transform) to map the pupil movable area onto the screen area. However, the main difference between our proposed gaze-tracking method and the previous methods [10,11] is that we used multi-geometric transform matrices (**T_1_**, **T_2_**, …, **T_4_**), whereas previous studies [10,11] used only a single geometric transform matrix to map the quadrangle defined by (*C_x1_*, *C_y1_*), (*C_x2_*, *C_y2_*), (*C_x3_*, *C_y3_*), and (*C_x4_*, *C_y4_*) into the rectangle defined by (*S_x1_*, *S_y1_*), (*S_x2_*, *S_y2_*), (*S_x3_*, *S_y3_*), and (*S_x4_*, *S_y4_*).

## 3. Experimental Results

**Figure 9.**
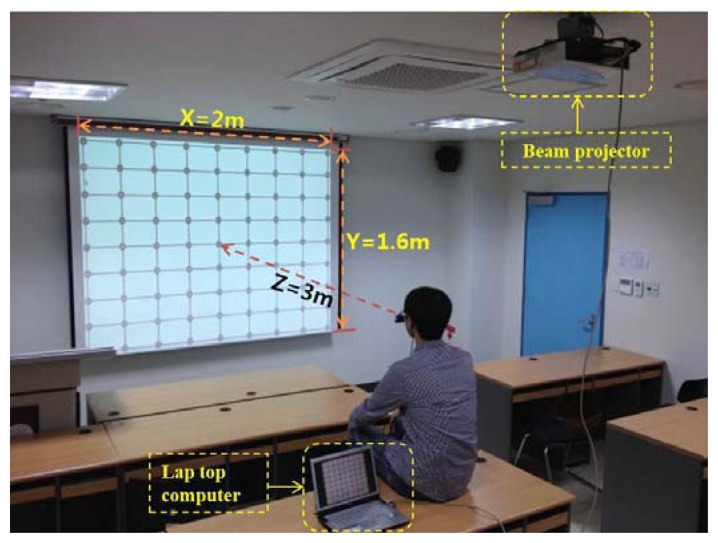
Experimental environment with a large screen.

The proposed gaze-tracking method was tested on a laptop computer with an Intel Core 2 Duo 1.83 GHz CPU and 1 GB RAM. The algorithm was developed in C++ using Microsoft Foundation Class (MFC), and the image capture software was produced using the DirectX 9.0 software development kit (SDK). In our experiments, each user gazed at 81 reference points on a screen, as shown in Figure 9. The screen size was 2 m &times 1.6 m (horizontal and vertical), and the distance from the user to the screen was approximately 3 m. Ten subjects participated in this experiment and each subject had six trials. Half of the data were used for training, and the other half were used for testing. This procedure was repeated by switching the training data and the testing data, and the average accuracy was calculated.

From the training data, we obtained the desired output positions for the MLP training. For example, we can train the MLP with the five desired output (virtual) points [(*C_x5_*, *C_y5_*), (*C_x6_*, *C_y6_*), (*C_x7_*, *C_y7_*), (*C_x8_*, *C_y8_*), and (*C_x9_*, *C_y9_*) in Figure 5], because these five points are the data acquired when user gazed at the positions (upper-center, middle-left, middle-center, middle-right, and lower-center positions of the screen in Figure 9) which were among the 81 gazing points acquired during the training procedure.

In the experiments, we measured the error of gaze detection (EGD) using Equation (10), where *Z* is the distance from the user's eye to the screen, *X_e_* is the error distance between the reference position and the calculated gaze position on the *x*-axis on the screen, and *Y_e_* is the error distance between the reference position and the calculated gaze position on the *y*-axis on the screen:

(10) $$\text{EGD}({}^{\circ})={\text{tan}}^{-1}\left(\frac{\sqrt{{{\text{X}}_{\text{e}}}^{2}+{{\text{Y}}_{\text{e}}}^{2}}}{\text{Z}}\right)$$

**Figure 10.**
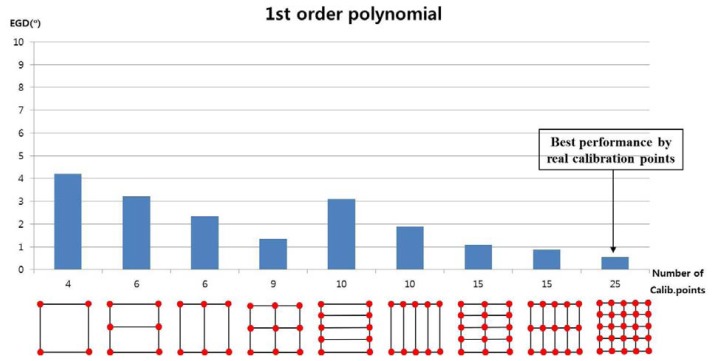
Error of gaze detection depending on the number of calibration points, when using a 1st-order polynomial mapping function.

We measured the EGD with increasing number of calibration points. In the first test, we used the 1st-order polynomial mapping function (geometric transform) in Equations (5) and (7). Figure 10 shows the performance with 4, 6, 9, 10, 15, and 25 calibration points. We applied geometric transform matrices to each subarea to map the pupil movable area onto the screen area. For example, when the number of calibration points was 9, a user actually gazed at nine calibration points. Four geometric transform matrices (**T_1_**, **T_2_**, **T_3_**, and **T_4_** in Figure 8) were used to calculate the gaze position in each sub-region. As shown in Figure 10, the EGD generally decreased as the number of calibration points increased, if the calibration points included the screen center. The EGD was lowest when a user gazed at 25 calibration points.

**Figure 11.**
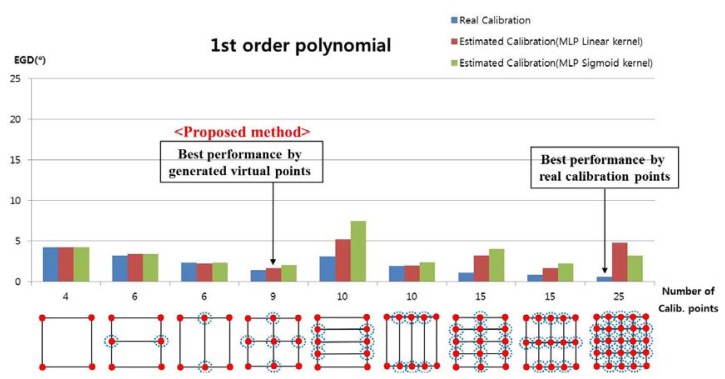
Error of gaze detection depending on the number of calibration points, when using the 1st-order polynomial function with “real calibration” (Figure 10) and the proposed method.

In the next experiment, we measured the EGD when using the proposed method to generate the virtual points with the 1st-order polynomial function, as shown in Figure 11. For example, with nine calibration points, each user actually gazed at four calibration points (the four corners of the screen, *i.e.*, the uncircled red points in Figure 11), and the virtual points (the red points inside blue dotted circles in Figure 11) were generated by the MLP algorithm, which used linear or sigmoid kernel functions. In Figure 11, “real calibration” refers to the results in Figure 10 (*i.e.*, where a user actually gazed at all of the calibration points without generating virtual points).

In most cases, the EGD with “real calibration” was less than that with the proposed method. However, the EGD with the proposed method was less than that with an existing method, when using four actual calibration points [11]. In a previous study [11], users gazed at a small viewing area and the calculated EGD was less than 1.6°. However, the larger area used in our research generated nonlinear movements of the pupil due to the greater rotation of the eyeball; therefore, the calculated EGD was > 4° (the extreme left bar in Figure 10) despite using the same method to calculate the gaze position [11].

When the proposed method generated five virtual points based on four actual points using MLP with a linear kernel, the EGD was less than that in other scenarios using the proposed method, as shown in Figure 11.

When the number of calibration points was ten (*i.e.*, six virtual points and four actual points), the EGD was higher than in other cases, as shown in Figure 11. The reasons for the higher EGD are as follows:

As shown in Figure 2 and Figure 9, the user gazed at a large display while the camera captured the user's eye image from below the eye. In addition, the horizontal length (2 m) of the display was longer than the vertical length (1.6 m). Thus, the nonlinear movement of the pupil was greater when the eye was rotated in the horizontal direction (*i.e.*, when a user gazed at the extreme upper or lower horizontal boundary of the display) than when the eye was rotated in the vertical direction (*i.e.*, when a user gazed at the extreme left or right horizontal boundary of the display). To compensate for the nonlinear movements of the pupil, points had to be generated for the extreme upper or lower boundary of the display. These points were not generated when the number of calibration points was 10, resulting in a higher EGD.

The EGD values for Figure 11 are shown in Table 2. With the proposed method (MLP with a linear kernel), the EGD was lowest (1.66°) in the scenario where the user actually gazed at four points and five additional virtual points were generated, compared to other scenarios.

When the user actually gazed at nine points, the EGD was 1.36°. The EGD was lowest when a user actually gazed at 25 calibration points (0.55°). Even with a higher EGD, the proposed method is much more convenient for the user, because they had to gaze at only four positions during the initial calibration stage. In addition, when the user actually gazed at four points, the EGD of the proposed method with five virtual points (1.66°) was much lower than the EGD of the existing method without virtual points (4.19°) [11].

**Figure 12.**
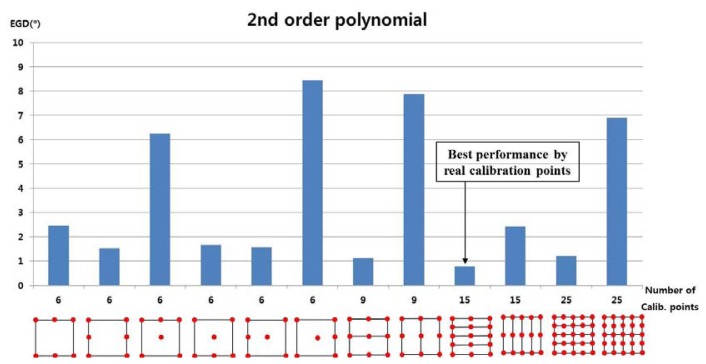
Error of gaze detection depending on the number of calibration points, when using a 2nd-order polynomial mapping function.

In the next test, we used the 2nd-order polynomial mapping function in Equations (6) and (8). In Figure 12, the numbers of calibration points were 6, 9, 15, and 25, and we applied the 2nd-order polynomial function to each subarea to map the pupil movable area onto the screen area. For example, when the number of calibration points was nine, the user actually gazed at nine calibration points and two 2nd-order polynomial functions were used to calculate the gaze position in two sub-regions. As shown in Equations (6) and (8), the 2nd-order polynomial function had 12 unknown parameters, and at least six calibration points were required to obtain those parameters. When the number of calibration points was nine, only two 2nd-order polynomial functions were defined, as shown in Figure 12. However, as shown in Equations (5) and (7), 1st-order polynomial function had eight unknown parameters, and at least four calibration points were required to obtain those parameters. With nine calibration points, the four 1st-order polynomial functions were defined as shown in Figure 10.

The experiment results showed that the EGD was lowest when a user actually gazed at 15 calibration points, as shown in Figure 12.

**Figure 13.**
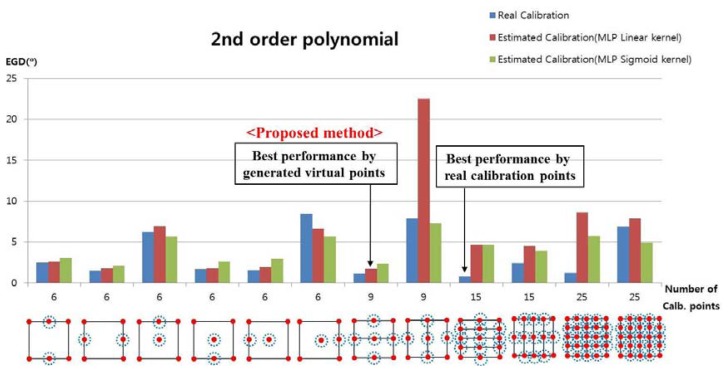
Error of gaze detection depending on the number of calibration points, when using the 2nd-order polynomial function with “real calibration” (Figure 10) and the proposed method.

In the next experiment, we measured the EGD when using the proposed method with the 2nd-order polynomial function to generate the virtual points, as shown in Figure 13. For example, with nine calibration points, each user actually gazed at four calibration points (the four corners of the screen, *i.e.*, the uncircled red points), and the virtual points (the red points inside the blue dotted circles) were generated with the MLP algorithm, which used linear or sigmoid kernel functions. In Figure 13, “real calibration” refers to Figure 12, where the user actually gazed at all of the calibration points without generating virtual points.

In most cases, the EGD with “real calibration” was lower than that with the proposed method. When the proposed method was used to generate five virtual points based on four actual points with the MLP using the linear kernel, the EGD was lower than that in other cases with the proposed method, as shown in Figure 13.

The EGD values for Figure 13 are shown in Table 3. With the proposed method (MLP with a linear kernel), the EGD was lowest (1.75°) in the scenario where the user actually gazed at four points and five additional virtual points were generated, compared to other scenarios. The EGD was lowest when a user actually gazed at 15 calibration points (0.78°); however, the proposed method is much more convenient for the user, because they had to gaze at only four positions during the initial calibration stage. The lowest EGD of the 2nd-order polynomial function (1.75°) was higher than the lowest EGD of the 1st-order polynomial function (1.66°), as shown in Table 2. Thus, we confirmed that the accuracy was better when using the 1st-order polynomial function.

The performance of the 2nd-order polynomial-based mapping function is worse than that of the 1st-order, because of the following reasons:

The lowest EGDs of both 1st-order and 2nd-order polynomial functions were obtained with five virtual points based on four actual (gazing) points. However, with the 2nd-order polynomial-based mapping function, two transform matrices were defined, as shown in Figure 13. On the other hand, with the 1st-order polynomial function, four transform matrices were defined, as shown in Figure 11. That is, twice as many transform matrices were used with the 1st-order polynomial function on a smaller pupil movement area; therefore, the correlation between the pupil movement area and the screen region can be more accurately (minutely) defined (Figure 8), thereby reducing the gaze-detection error.

As shown in the Equations (6) and (8), six points are required for determining one 2nd-order polynomial function because the number of unknown parameters is 12 [*a*, *b*, … *l* of Equations (6) and (8)]. However, only four points are required for determining one 1st-order polynomial function because the number of unknown parameters is eight [*a*, *b*, … *h* of Equations (5) and (7)]. So, the only two matrices are obtained for the 2nd-order polynomial function in the 1st case that “Number of Calib. points” is nine in the Figure 13. But the four matrices are obtained for the 1st-order polynomial function in the case that “Number of Calib. points” is nine in the Figure 11.

However, the comparisons of the 2nd-order and the 1st-polynomial functions were also made with the same condition, *i.e.*, using four transformation matrices for the both cases. As shown in the two cases that “Number of Calib. points” is 15 in the Figure 13, the four transform matrices are used for the 2_nd_-order function in the both cases, respectively. In these cases, the EGDs with MLP with linear kernel are 4.68° and 4.5°, respectively, as shown in Table 3, which are larger than the EGD (1.66°) by the 1st-order polynomial function with MLP with linear kernel and the four transformation matrices as shown in Table 2. In addition, the EGDs with MLP with sigmoid kernel are 4.65° and 3.9°, respectively, as shown in Table 3, which are larger than the EGD (2.04°) by the 1st-order polynomial function with MLP with sigmoid kernel and the four transformation matrices as shown in Table 2.

**Figure 14.**
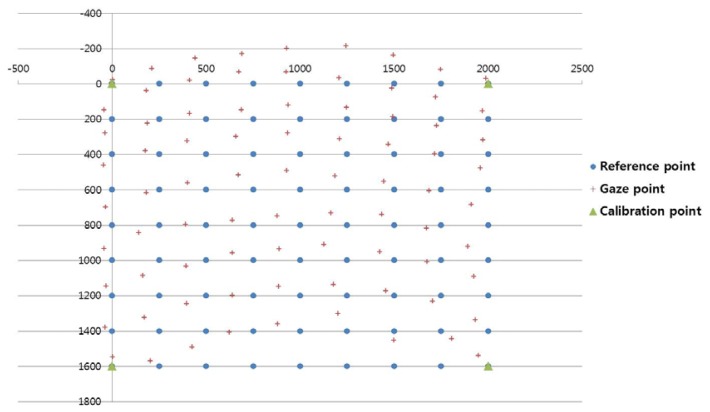
Example of the gaze points calculated using the existing method [11], which required the user to gaze at four calibration points.

**Figure 15.**
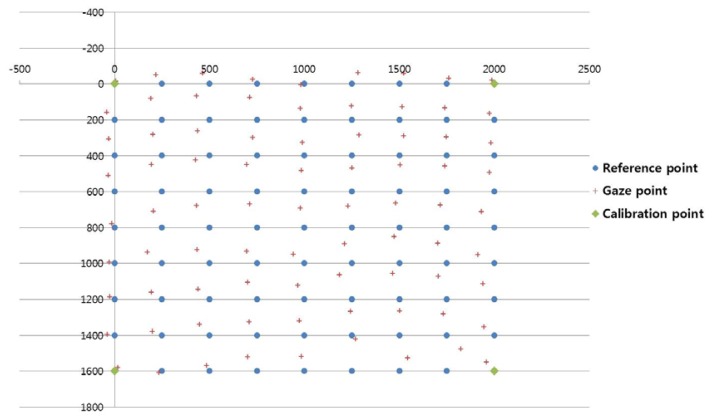
Example of the gaze points calculated using the proposed method, which required the user to gaze at four calibration points and which generated five virtual points using MLP with a linear kernel and the 1st-order polynomial function.

**Figure 16.**
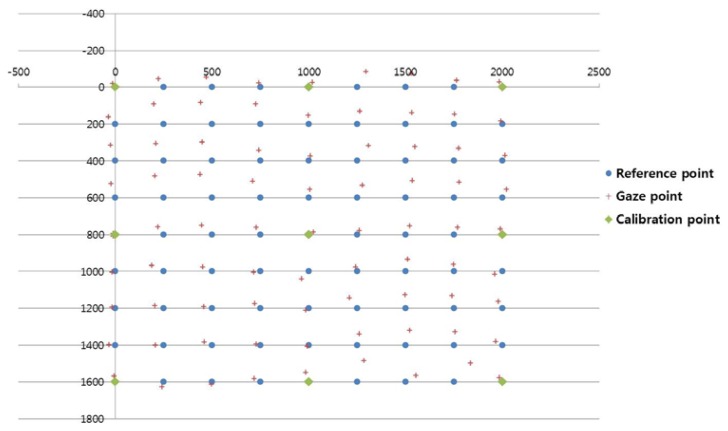
Example of the gaze points calculated using the “real calibration” method (in Table 1 and 2), which required the user to gaze at nine calibration points.

Figure 14, Figure 15, and Figure 16 show examples of the experiment results. In Figure 14, the user gazed at four calibration points, and the gaze position was calculated using the existing geometric method without generating virtual points [11]. Figure 15 shows the results using the proposed method with the lowest EGD in Table 2. The same user gazed at the four calibration points, and five virtual points were generated using MLP with a linear kernel. The gaze position was calculated based on the 1st-order polynomial function. Figure 16 shows the results with the “real calibration” method using the lowest EGD in Table 2. The same user gazed at nine calibration points, and the gaze position was calculated using the multi-geometric transform method.

The proposed method (Figure 15) was less accurate than the “real calibration” method (Figure 16); however, the proposed method was much more convenient to use, because fewer points were needed for the initial calibration. In addition, the proposed method was more accurate than the existing method (Figure 14).

In the final experiment, we measured the processing time with the proposed gaze-tracking method. Detecting the pupil center took 16 ms, generating new calibration points required 1 ms, and calculating the final gaze position took 20 ms. Thus, the total processing time was approximately 37 ms, and we confirmed that the processing speed with the proposed method was approximately 27 fps.

## 4. Conclusion

In this paper, we proposed a new gaze-tracking method to improve the performance of a gaze-tracking system using a large screen at a distance. The proposed device was light and wearable, and it was comprised of a USB camera, a zoom lens, and an NIR-LED. The proposed method generated five virtual points using an MLP with a linear kernel based on four actual points (detected pupil centers) as the input. The five virtual points and four actual points were used in multi-geometric transforms to calculate the final gaze position. The proposed system is more accurate and more convenient to use than the existing method, because it requires fewer calibration points.

In future work, we will test the proposed method in various environments, such as gaze detection on the small display of a mobile device or gaze detection while driving a vehicle. In addition, we would research a method that hides the calibration process from the users; for example, by requesting a user to watch a moving target on the screen, while the system acquires the data points needed for calibration.

## Figures and Tables

**Table 1.** Number of calibration points in the previous and proposed gaze-tracking methods

**Number of Calibration Points**	**#points<5**				**5 ≤ #points<20**					**20 ≤ #points**		

	**0**	**1**	**2**	**4**	**5**	**8**	**9**	**12**	**16**	**25**	**42**	**200**
Gaze-Tracking Method	[12]	[5], [6], [8], [10], [13]	[14], [15]	[4,7], [16], and **proposed** **method**	[17]	[18]	[19], [20], [21]	[22]	[23]	[3]	[24]	[25]

**Table 2.** Comparison of EGD results in Figure 11 (1st-order polynomial function) (unit:°)

**Method**	**Number of Calibration Points**								

	**4**	**6**	**6**	**9**	**10**	**10**	**15**	**15**	**25**
Real Calibration	4.19	3.21	2.33	1.36	3.1	1.89	1.09	0.88	**0.55**
**Proposed Method (MLP with Linear Kernel)**	4.19	3.38	2.23	**1.66**	5.23	1.98	3.21	1.7	4.79
Proposed Method (MLP with Sigmoid Kernel)	4.19	3.38	2.35	2.04	7.45	2.37	4.03	2.19	3.23

**Table 3.** Comparison of the EGD results in Figure 13 (2nd-order polynomial function) (unit:°)

Number of Calibration Points	6	6	6	6	6	6	9	9	15	15	25	25
Method												
Real Calibration	2.46	1.52	6.26	1.66	1.57	8.44	1.13	7.87	**0.78**	2.43	1.2	6.9
**Proposed Method (MLP with Linear Kernel)**	2.6	1.79	6.96	1.77	1.95	6.63	**1.75**	22.52	4.68	4.5	8.6	7.86
Proposed Method (MLP with Sigmoid Kernel)	3.06	2.05	5.65	2.6	2.96	5.67	2.35	7.3	4.65	3.9	5.75	4.9
